# Mitochondrial pyruvate dehydrogenase phosphatase metabolism disorder in malignant tumors

**DOI:** 10.32604/or.2025.063716

**Published:** 2025-07-18

**Authors:** YUFENG WANG, HUIFENG DANG, QIANQIAN WANG, SHUXIAO WU, LEI HAN, XU LUO, YINGXIA TIAN, HAILIN TANG

**Affiliations:** 1 Department of Breast Medical Oncology, Affiliated Cancer Hospital of Sun Yat-sen University, Gansu Hospital, Lanzhou, 730050, China; 2 State Key Laboratory of Oncology in South China, Guangdong Provincial Clinical Research Center for Cancer, Sun Yat-sen University Cancer Center, Guangzhou, 510060, China

**Keywords:** Malignant tumors, Mitochondria, Pyruvate dehydrogenase phosphatase (PDP), Metabolism

## Abstract

This review focuses on the metabolic issues related to mitochondrial pyruvate dehydrogenase phosphatase (PDP) in malignant tumors and its potential mechanisms. Recent research on tumor metabolic mechanisms has shown that PDP dysregulation is closely linked to metabolic reprogramming in tumor cells, and potentially promotes tumor. Research has comprehensively explored the structural-functional characteristics of PDP, its metabolic regulatory mechanisms, and its role in various types of malignant tumors. Nevertheless, several questions still exist regarding its potential mechanisms within acetylation, phosphorylation, hypoxia, immune infiltration, mitochondrial metabolism, drug resistance, oxidative phosphorylation, and tumor prognosis. This article intends to summarize the latest research, examine PDP’s potential as a therapeutic target, and propose future research directions to enhance cancer treatment strategies.

## Introduction

In recent years, the global incidence of malignant tumors has risen alarmingly [1], posing a significant public health challenge [2,3]. With a deeper understanding of tumor biology, researchers are increasingly focusing on changes in tumor cell metabolism, a significant area of study. PDP, a key metabolic enzyme, plays a crucial role in altering cellular energy metabolism [4]. Research indicates that abnormal PDP activity is closely associated with the development of various malignant tumors [5,6]. This is particularly evident in the metabolic balance between glycolysis and oxidative phosphorylation (OXPHOS) within tumor cells [7]. Specifically, the pyruvate dehydrogenase complex (PDC) links glycolysis to the tricarboxylic acid cycle, catalyzing the oxidation of pyruvate to produce acetyl-CoA, which is crucial for cellular energy metabolism [8]. In tumor cells, PDC activity is regulated by multiple factors, with the interaction between pyruvate dehydrogenase kinase (PDK) and PDP being crucial for metabolic reprogramming [9]. Even in the presence of oxygen or a fully functional mitochondrial respiration chain, tumors undergo the oxidation of glucose into lactic acid, known as the “Warburg effect”, which is a hallmark of cancer cell metabolism and reflects their unique metabolic adaptability [10]. However, in mitochondria, high PDK expression inhibits PDC activity, leading tumor cells to adopt a high glycolytic phenotype [11]. Recent studies have also highlighted the important role of PDP in regulating tumor cell energy metabolism, particularly in adapting to hypoxic and nutrient-deprived conditions within the tumor microenvironment, driving in-depth research on this enzyme [12]. Thus, a deeper understanding of the specific mechanisms of PDP in tumor development not only clarifies the complexity of cancer metabolism but also identifies potential targets and therapeutic avenues for new treatment strategies.

### Abnormal glycolytic pathways in tumor cells

Based on the “Warburg effect” theory, recent tumor-related research has focused on the phenomena, mechanisms, and consequences of metabolic changes in malignant tumor cells [13,14]. The metabolic profile of cancer cells differs significantly from that of non-tumor cells. Since all tumors rely on metabolic alterations to support growth, metastasis, and survival, these atypical metabolic pathways may be potential targets for anti-tumor drugs [15,16]. Carbohydrate metabolism serves as the primary pathway for adenosine triphosphate (ATP) production and other bio-molecular syntheses in cells [17]. Cancer cells metabolize glucose differently from normal cells, with rapidly proliferating cancer cells predominantly obtaining ATP through aerobic glycolysis instead of relying on OXPHOS [18,19]. During glucose metabolism, most pyruvate is reduced to lactate in the cytoplasm rather than being transported to mitochondria [20].

**Figure 1:**
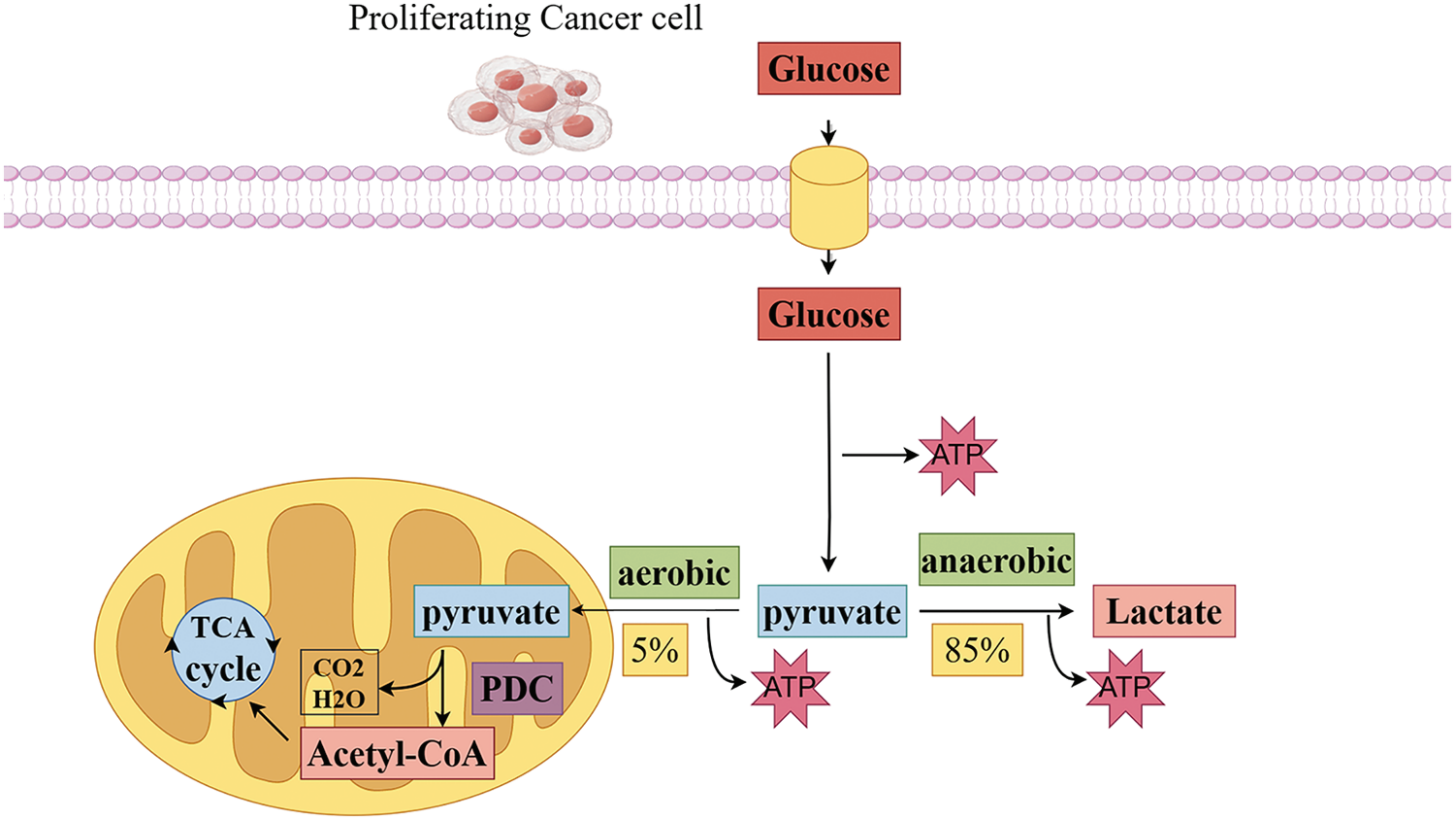
Pyruvate is a key point in regulating energy metabolism in tumor cells. The glycolytic pathway begins glucose metabolism by breaking down glucose into pyruvate. In anaerobic conditions, approximately 85% of pyruvate is converted to lactate in the cytoplasm by lactate dehydrogenase, which maintains a continuous energy supply from glycolysis. Conversely, in aerobic conditions, approximately 5% of pyruvate enters the mitochondrial matrix, There, it undergoes oxidative decarboxylation by pyruvate dehydrogenase to form acetyl-CoA. and then enters the tricarboxylic acid cycle and interacts with the electron transport chain for efficient energy conversion (Conducted by Figdraw).

In the process of tumor cell development, mitochondria play a role in various transformations of tumor cell characteristics, including abnormal energy metabolism, resistance to cell death, tissue invasion and metastasis, promotion of tumor inflammation, genomic instability, and escape from immune killing [21]. Changes in cellular energy metabolism are crucial for tumor growth [22], and other tumor biological characteristics are closely related to mitochondrial metabolic functions [23]. Pyruvate, the final product of glycolysis, is essential for various cellular processes, including oxidative metabolism, gluconeogenesis, lipid and cholesterol synthesis, and maintaining tricarboxylic acid (TCA) cycle flux. Under aerobic conditions, tumor cells prefer to convert pyruvate into lactate rather than enter mitochondrial oxidative metabolism. This preference indicates a strong connection between mitochondrial pyruvate metabolic abnormalities, defects in regulatory enzymes, and the development and metastasis of tumors [24]. The pyruvate dehydrogenase and pyruvate carboxylase required for pyruvate oxidative metabolism are only present in the mitochondrial matrix. Thus, cytoplasmic pyruvate undergoes dehydrogenation and carboxylation to enter the mitochondrial matrix, driving the TCA cycle and enabling OXPHOS and ATP production, ultimately facilitating multiple biosynthetic metabolic processes [25]. Therefore, controlling the fate of pyruvate in cells is a key point in regulating energy metabolism (Fig. 1).

### Mitochondrial pyruvate transport disorders and malignant tumors

Pyruvate metabolism is a central component of carbon homeostasis, an evolutionarily conserved process. Abnormalities in this metabolism are associated with various human metabolic disorders, contributing to diseases like cancer. Pyruvate, a glycolysis product, is primarily generated in the cytoplasm through glucose glycolysis [26] and is then transported to mitochondria via voltage-dependent anion channels (VDAC) or porin proteins for further metabolic processes [27,28]. Pyruvate entry into the mitochondrial matrix is essential for the generating of reducing equivalents and ATP, as well as for biosynthesizing glucose, fatty acids, and amino acids from pyruvate. Pyruvate’s entry into the mitochondrial matrix is facilitated by specific mitochondrial pyruvate carriers (MPC). MPC facilitates the transport of cytoplasmic pyruvate (and a proton) across the inner mitochondrial membrane (IMM), linking glycolysis to OXPHOS. In cells with high mitochondrial content, the majority of pyruvate generated by glycolysis is transported into mitochondria for further metabolic processes [29,30]. Loss of MPC gene expression or reduced MPC activity results in decreased pyruvate oxidative metabolism in tumor cells. Additionally, factors such as pyruvate concentration, redox state, and post-translational regulation of MPC activity influence MPC flux [31,32].

Bricker et al. and Herzig et al. confirmed the composition of human MPC, which consists of MPC1 and MPC2 (formerly Brp44L and Brp44). This highly conserved heterodimer is essential for maintaining stability and facilitating pyruvate transport [33,34]. The human MPC heterodimer exhibits higher binding affinity for substrates and inhibitors. It mediates pyruvate transport across mitochondrial membranes and is the main functional unit for this process [35,36]. The mitochondrial pyruvate carrier is situated at the crossroads of glycolysis and mitochondrial pyruvate metabolism. In most tumor cells, glycolysis is enhanced, while OXPHOS is reduced [37,38]. Therefore, MPC should play a crucial pathophysiological role in metabolic alterations in tumor cells [39]. The “Warburg effect” is also seen as arising from reduced or lost MPC function [40]. Research indicates that MPC proteins are often downregulated or insufficiently expressed in various cancers, potentially influencing the “Warburg effect” through their regulatory functions [41–43]. The regulation of MPC varies across different cancer types and tumor cell types. MPC1 gene expression is downregulated in most tumor types, excluding hematologic malignancies, leukemia, and lymphoma. Low MPC1 expression is associated with a poor prognosis in esophageal, gastric, colorectal, kidney, lung, and prostate cancers [44–47]. MPC2 expression varies in this group of solid tumors, with some showing high levels and others low levels [48]. Low expression is specifically linked to poor prognosis in kidney and colorectal cancers [49–51]. *In vitro* experiments further validate this scientific perspective [52]. In breast cancer cells, MPC1 directly targets estrogen-related receptors [53]. In hepatocellular carcinoma cells, the pro-apoptotic gene PUMA is induced by p53 and disrupts the oligomerization and function of MPC at the post-translational level. This disruption illustrates how mediators of cancer metabolism and proliferation can regulate mitochondrial metabolism by modulating MPC activity [54]. Clinical studies indicate that MPC expression affects cancer treatment efficacy; in hepatocellular carcinoma, inhibiting the peroxisome proliferator-activated receptor gamma coactivator 1 alpha (PGC1α)/nuclear respiratory factor (NRF1)-MPC axis increases sensitivity to sorafenib and doxorubicin [46]. The absence of the MPC1 gene causes cancer cell lines, specifically those from esophageal squamous cell carcinoma, prostate cancer, and pancreatic cancer, to resist radiotherapy and chemotherapy [41,42,55]. Patients expressing low levels of MPC1 have lower survival rates than those expressing high levels of MPC1 [56]. Additionally, overexpressing MPC reduces cancer stemness, which suggests that a decrease in MPC activity and reduced pyruvate entry into the TCA cycle are necessary for cancer cells to manifest their carcinogenic effects [55,57]. Furthermore, MPC inactivation significantly contributes to cancer cell proliferation, invasion, migration, epithelial-mesenchymal transition (EMT), and extracellular matrix (ECM) remodeling, highlighting its crucial role in cancer metastasis [58,59]. Research results also show that both MPC1 and MPC2 are low in prostate cancer, positively correlated with prognosis, and the loss of their expression results in more aggressive tumor cells. Inhibition of MPC1 expression increases glycolytic metabolism in prostate cancer cells and promotes tumor malignancy [48,60]. After clarifying the molecular characteristics of MPC, a biosensor (RESPYR) based on fluorescence resonance energy transfer (BRET) technology was designed to monitor MPC activity in real-time. Studies have found that MPC activity in tumor cells is very low, with a preference for using glycolysis to provide ATP. By increasing cytoplasmic pyruvate concentration, MPC activity can be enhanced, thereby increasing OXPHOS [61]. Additionally, inhibiting MPC activates fatty acid dehydrogenase and directs glutamine into the TCA cycle. Research has shown that Fabp4 plays a crucial role in promoting the progression of triple-negative breast cancer (TNBC) due to the tumors’ high lipid metabolic activity. It influences mitochondrial stability, facilitates CPT1-related fatty acid oxidation, and regulates the production of reactive oxygen species (ROS) [62].This increases the reliance of cancer cells on glutamine metabolism and the interaction of MPC1 with fatty acid dehydrogenase, significantly hindering tumor growth [52].

### Structure and function of pyruvate dehydrogenase phosphatase composition and activity regulation of pyruvate dehydrogenase phosphatase

PDP is a crucial enzyme that is primarily responsible for catalyzing the dephosphorylation of pyruvate dehydrogenase to effectively regulate its activity. PDP typically consists of a dimer formed by a catalytic subunit called PDP1 and a regulatory subunit known as PDP2; notably, PDP1 exhibits higher catalytic activity than PDP2. PDP activity is affected by different factors, including how it is phosphorylated, the concentration of substrates, and the presence of metal ions.

Studies have shown that PDP activity is crucial for cellular metabolism, particularly in energy production and glycolysis. By analyzing the crystal structure of PDP through 1.8A resolution in rats, the structure reveals that its catalytic site contains manganese ions, which may be essential for PDP catalytic activity [63]. Additionally, PDP activity is precisely regulated by multiple intracellular signaling pathways, including adenosine 5′-monophosphate (AMP)-activated protein kinase (AMPK) and mammalian target of rapamycin (mTOR), crucial for cellular energy balance and metabolic regulation, thus emphasizing the significance of PDP in cellular physiology [5].

Pyruvate dehydrogenase phosphatase (PDP) is an important and key enzyme primarily responsible for catalyzing the dephosphorylation and activation of the E1 component α subunit of the pyruvate dehydrogenase complex (PDC), thereby stimulating the mitochondrial enzyme that converts pyruvate to acetyl-CoA. PDP is typically composed of heterodimers encoded by the *PDP1* and *PDP2* genes, with PDP1 exhibiting higher catalytic activity than PDP2. PDP1 is the most studied phosphatase isoform, consisting of a catalytic subunit (PDP1c) and a regulatory subunit (PDP1r). PDP1 catalyzes the dephosphorylation of phosphorylated E1 (P-E1) that requires Mg^2+^ and Ca^2+^ stimulation by binding to the L2 (lipoyl) domain of the dihydrolipoamide acetyltransferase (E2) core in a Ca^2+^-dependent manner. During this process, PDP1r regulates the conformational change of PDP1c, and it has been found that as the Mg^2+^ concentration increases, the sensitivity of PDP1c to Ca^2+^ also increases. When PDP1c binds to PDP1r, PDP1c becomes sensitive to Mg^2+^ levels, and the binding of PDP1c to L2 creates a tight Ca^2+^ binding site. The PDP1r subunit facilitates the connection between this site and the Mg^2+^ site on PDP1c, making both sites sensitive to spermine, and thereby participating in tumor metabolic reprogramming. Studies utilizing sedimentation velocity and equilibrium experiments show that PDP1c exists as a reversible mixture of monomers and dimers. The L2 domain preferentially binds to the PDP1c monomer, forming a complex with tightly bound Ca^2+^. This binding further reduces the likelihood of PDP1c forming dimers [64]. The biological activity of PDP proteins is complexly regulated by various factors, including but not limited to phosphorylation status, substrate concentration, and the influence of metal ions. Research shows that the activity of PDP plays a crucial role in cellular metabolism, particularly in energy metabolism and glycolytic pathways, making it indispensable. Further analysis through the Uniprot database confirms that PDP carries two cofactors, Mn^2+^ and Mg^2+^, with each subunit binding two Mn^2+^ and two Mg^2+^, where Mn^2+^ can replace Mg^2+^ in catalytic activity. Mn^2+^ primarily binds at the 144, 145, 418, and 516 amino acid sites of the PDP1 protein and at the 141, 142, 412, and 508 amino acid sites of the PDP2 protein. Additionally, the activity of PDP is finely regulated by multiple intracellular signaling pathways, such as the AMPK and mTOR pathways, which play key roles in cellular energy balance and metabolic regulation, further highlighting the importance of PDP in cellular physiology [65].

### Key enzymes in regulating pyruvate metabolism

**Figure 2:**
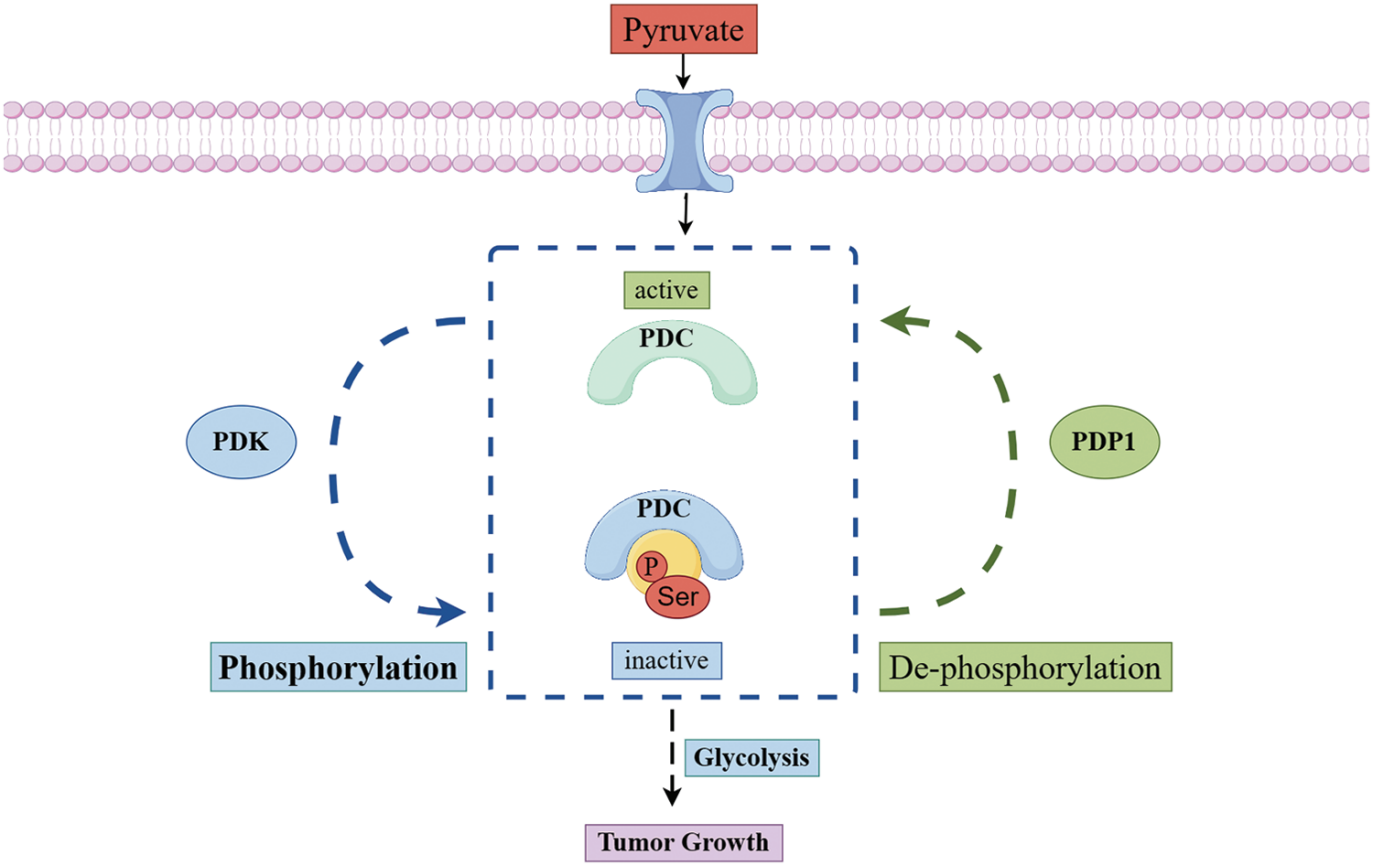
The metabolic mechanism of pyruvate dehydrogenase in mitochondrial. Once pyruvate enters the mitochondria, the reversible phosphorylation of the pyruvate dehydrogenase complex regulates its metabolism. Phosphorylation of PDC by PDK decreases its activity, which reduces the oxidative metabolism of pyruvate. Conversely, when PDP removes the phosphorylation from PDC, it reactivates the complex, increases pyruvate oxidative metabolism (Conducted by Figdraw).

Once pyruvate enters the mitochondrial matrix, it is irreversibly converted by the PDC along with nicotinamide adenine dinucleotide (NAD+) into three products: acetyl-CoA, NADH, and carbon dioxide. Acetyl-CoA then either participates in the TCA cycle or regulates the synthesis of cholesterol, fats, and acetylcholine. The primary components of PDC are pyruvate dehydrogenase (E1), dihydrolipoyl transacetylase (E2), dihydrolipoyl dehydrogenase (E3), and E3-binding protein (E3BP). Additionally, PDC activity is regulated by two enzymes: PDK and PDP [66–68]. The primary regulatory mechanism of mammalian PDC is the reversible phosphorylation of three specific serine residues on the E1α subunit: ser293 (site 1), ser300 (site 2), and ser323 (site 3) [69]. PDK can phosphorylate the E1α subunit of PDC, resulting in reduced PDC activity [70]. As a result, pyruvate oxidative metabolism decreases, cytoplasmic lactate production increases, and acetyl-CoA production is diminished [71]. In contrast, PDP counteracts the effects of PDK by dephosphorylating pyruvate dehydrogenase E1 component subunit alpha (PDCE1a). This action reactivates PDC, increases the production of acetyl-CoA, and supports either OXPHOS or biosynthesis pathways [7]. In contrast to other protein complexes, which often exhibit cancer-related changes, there is barely any evidence of such changes in pyruvate dehydrogenase (PDH) gene expression, nor is there sufficient evidence for different subtypes or splicing variants of PDH complexes in cancer cells [72]. Additionally, the regulation of pyruvate metabolism is closely related to the interaction between lactate dehydrogenase (LDH) and other various metabolic enzymes, which together significantly affect the overall metabolic pathway and energy balance of tumor cells. Studying PDP is vital for understanding how it affects glucose metabolism and interacts with signaling molecules and transcription factors. This knowledge helps us see changes in cellular metabolism (Fig. 2).

### Mitochondrial metabolism in cellular energy balance

Mitochondria are often referred to as the powerhouse of cellular energy metabolism, and their functions go beyond mere ATP synthesis to include the precise regulation of various complex metabolic pathways. Mitochondria are often called the powerhouse of cellular energy metabolism, Their functions go beyond ATP synthesis and include precise regulation of various complex metabolic pathways. Research has demonstrated that mitochondrial function is influenced by several factors, including nutritional status, oxygen concentration, and the interplay of cellular signaling pathways [73]. Furthermore, changes in mitochondrial calcium ion concentration have been demonstrated to significantly influence energy metabolism. Calcium ions regulate the mitochondrial respiratory chain and energy production, which are essential for the fine-tuning of cellular metabolism [74]. Mitochondrial biogenesis and dynamic changes, including fusion and fission, are essential for maintaining cellular energy balance. Dysregulation of these processes can lead to various metabolic diseases. Mitochondrial DNA mutations, which affect the respiratory chain, reduce OXPHOS efficiency and further promote tumor cells’ dependence on specific metabolic pathways [75]. The activation of mitochondrial stress response mechanisms in tumor cells, coupled with mitochondrial dysfunction, plays a crucial role in facilitating their growth and metastasis [76]. In prostate cancer, the burden of mitochondrial DNA mutations is closely related to the malignancy of the tumor and patient prognosis [77]. By changing the intracellular redox state, mitochondrial DNA mutations can induce metabolic reprogramming, which further promotes tumor progression and metastasis [78]. The accumulation of mitochondrial DNA mutations has been found to be closely linked to genomic instability in cancer cells, a significant feature in the development of tumors [79]. The mitochondrial stress response profoundly affects tumor cell metabolism and growth, significantly influencing invasiveness and treatment resistance. Mitochondrial dysfunction can cause elevated levels of intracellular reactive oxygen species (ROS), which can further promote tumor growth and metastasis [80]. Thus, by deepening our understanding of mitochondrial metabolism, we not only clarify the complexities of energy regulation but also uncover essential insights into the mechanisms of related diseases.

### Pyruvate dehydrogenase phosphatase in malignant tumors

In 2000, Hanahan and Weinberg defined six fundamental characteristics of malignant tumors: they exhibit growth independence, continuous proliferation, resistance to apoptosis, unlimited replication, sustained angiogenesis, and the ability to invade tissues and metastasize [81]. In 2022, Hanahan proposed four new characteristics of malignant tumors: immune evasion, abnormal cellular energy metabolism, genomic instability with mutations, and the promotion of tumor inflammation [82]. Among them, metabolic abnormalities in tumor cells are listed as a major feature of tumor tissue, and although the genetic background varies, the “Warburg effect” is widely present in various tumor cells, considered a characteristic of tumor cells. PDP, a crucial phosphatase that governs glycolysis and OXPHOS, impacts aerobic glycolysis and notably alters tumor cell metabolism (Table 1).

**Table 1:** The research of pyruvate dehydrogenase phosphatase in malignant tumors

Gene subtype	Research	Tumor	Research contents	Research methods	Key results
**PDP1**	Fan et al. [85]	Lung cancer	Acetylation	Cell line	ACAT1 enhances glycolysis by regulating both PDP1 and PDHA1. At the same time, SIRT3 collaborates with ACAT1 to influence the deacetylation of PDHA1 and the acetylation of PDP1.
	Shan et al. [86]	Pan-cancer	Phosphorylation	Cell line	Phosphorylation at the Y381 site of PDP1 changes ACAT1 and PDP1 expression, regulates PDP1’s lysine acetylation, and impacts tumor cell metabolism and proliferation.
					Phosphorylation of Tyr-94 on PDP1 decreases its activity by impairing its ability to bind lipoate and dihydrolipoamide acetyltransferase.
	Karagiota et al. [87]	Pan-cancer	Phosphorylation	Cell line	Under hypoxic conditions, the consumption of PDP1 influences the binding and acetylation of HIF-1 to specific gene promoters.
					When both PDPK1 and PDH are overactivated in such conditions, they enhance HIF-1 activit, promote PDK1 expression and decreasing the conversion of pyruvate to acetyl-CoA, thus facilitating glycolysis.
	Chen et al. [97]	Breast cancer	Immunoinfiltration	Bioinformatics	High expression of PDP1 is negatively correlated with CD8+ T cell infiltration in breast cancer.
	Li et al. [98]			Breast cancer cell	
				Macrophage	Notch1 increases PDP1 expression, mediating PDH activation and enhancing glycolysis.
	Giorgi et al. [99]	Glioblastoma	Mitochondrial metabolism	Genomics	Defects in mitochondrial complex I reduce the levels of PDH in both the cytoplasm and nucleus via the Ca^2+^-PDP1-PDH pathway, this decrease promotes DNA damage repair responses by decreasing histone acetylation.
				Cell line	
	Soriano-Baguet et al. [100]				
	Shi et al. [101]				
					Inhibition of PDH activity, caused by downregulation of PDP expression through the RAS-dependent signaling pathway, is related to the decreased reserve capacity of mitochondria.
	Alshamleh et al. [114]	Malignant myeloid leukemia	Drug resistance	FLT3-ITD+ cell	After PDP1 knockdown, cellular respiration declines, impairing the growth of FLT3-ITD positive cells.
					Under hypoxic conditions, FLT3-ITD positive cells continue to rely on PDP1.
	Liu et al. [115]	Lymphoma	Oxidative phosphorylation	Cell line	EGR1 mediates the activation of PDP1, increasing the production of intracellular ATP, reprogramming to OXPHOS, and generating sufficient energy.
	Song et al. [116]	Ovarian cancer	Tumor prognosis	Bioinformatics	PDP1 promotes the proliferation, invasion, and migration of ovarian cancer cells and is associated with poor prognosis.
	Wang et al. [117]	Breast cancert		Tissues and cell line	
	Li et al. [5]	Pancreatic cancer		Tissue	PDP1 gene is overexpressed in breast cancer tissues and correlates with poor prognosis, Validation through *in vivo* and *in vitro* experiments further confirmed that PDP1 contributes to cancer progression by regulating STAT3 phosphorylation levels.
	Chen et al. [6]	Prostatic cancer		Tissues	
	Deng et al. [121]	Low-grade gliomas		Single Cell transcriptome sequencing	PDP1 increases ATP production and promotes pancreatic cancer cell proliferation, invasion, and migration.
		Head and neck squamous cell carcinoma			High expression of PDP1 is associated with a poor prognosis in prostate cancer.
		Pancreatic cancer			Hsa-miRNA-655-3p and Hsa-miRNA-135b-5p positively regulate of PDP1
					Hsa-miRNA-135b-5p negtively regulate of PDP1
**PDP2**	Zhu et al. [125]	Breast cancer		Tissues	PDP2 expression is associated with the prognosis of breast cancer, and PDP2 dephosphorylation inhibits ACSL4 activity, inducing ferroptosis in tumor cells.
	Rellinger et al. [126]	Neuroblastoma		Cell line	GRP-R regulates glucose metabolism in neuroblastoma by modulating HIF-1α, PDK4, and PDP2.
	Chen et al. [127]	Prostatic cancer		CTC detection	PDP2 is associated with the prostate cancer metastatic process.
	Wang et al. [128]	Lung cancer		Cell line	After induction by ML-ESPS, PDP2 is significantly overexpressed.

### Pyruvate dehydrogenase phosphatase catalytic subunit 1 in malignant tumors

**Figure 3:**
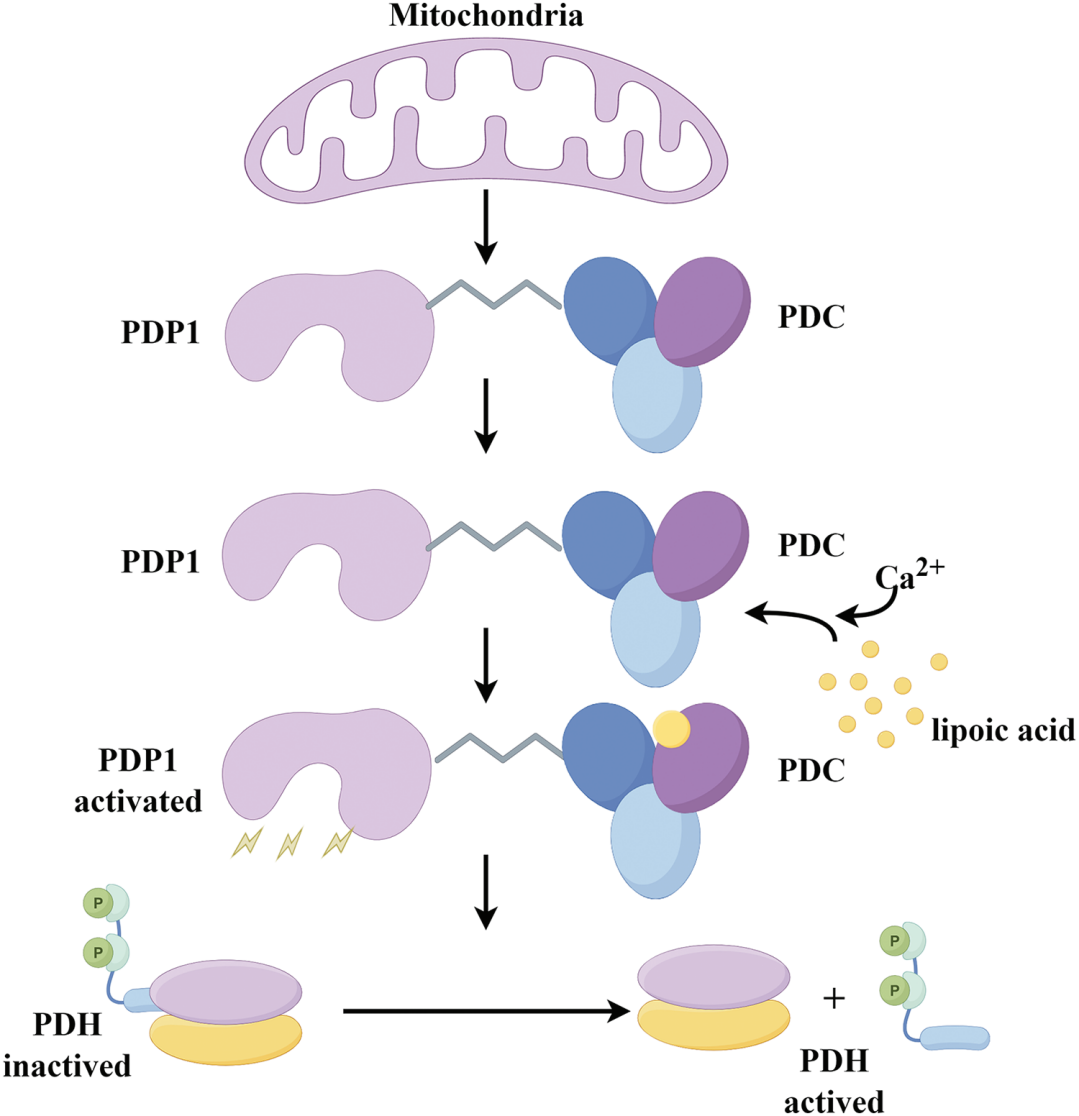
The mechanism diagram provides a detailed overview of PDP1 phosphatase activity in mitochondrial. By binding to the L2 domain of the PDC E2 subunit in a Ca^2+^-dependent manner, PDP1 catalyzes the dephosphorylation of PDH (Conducted by Figdraw).

**Figure 4:**
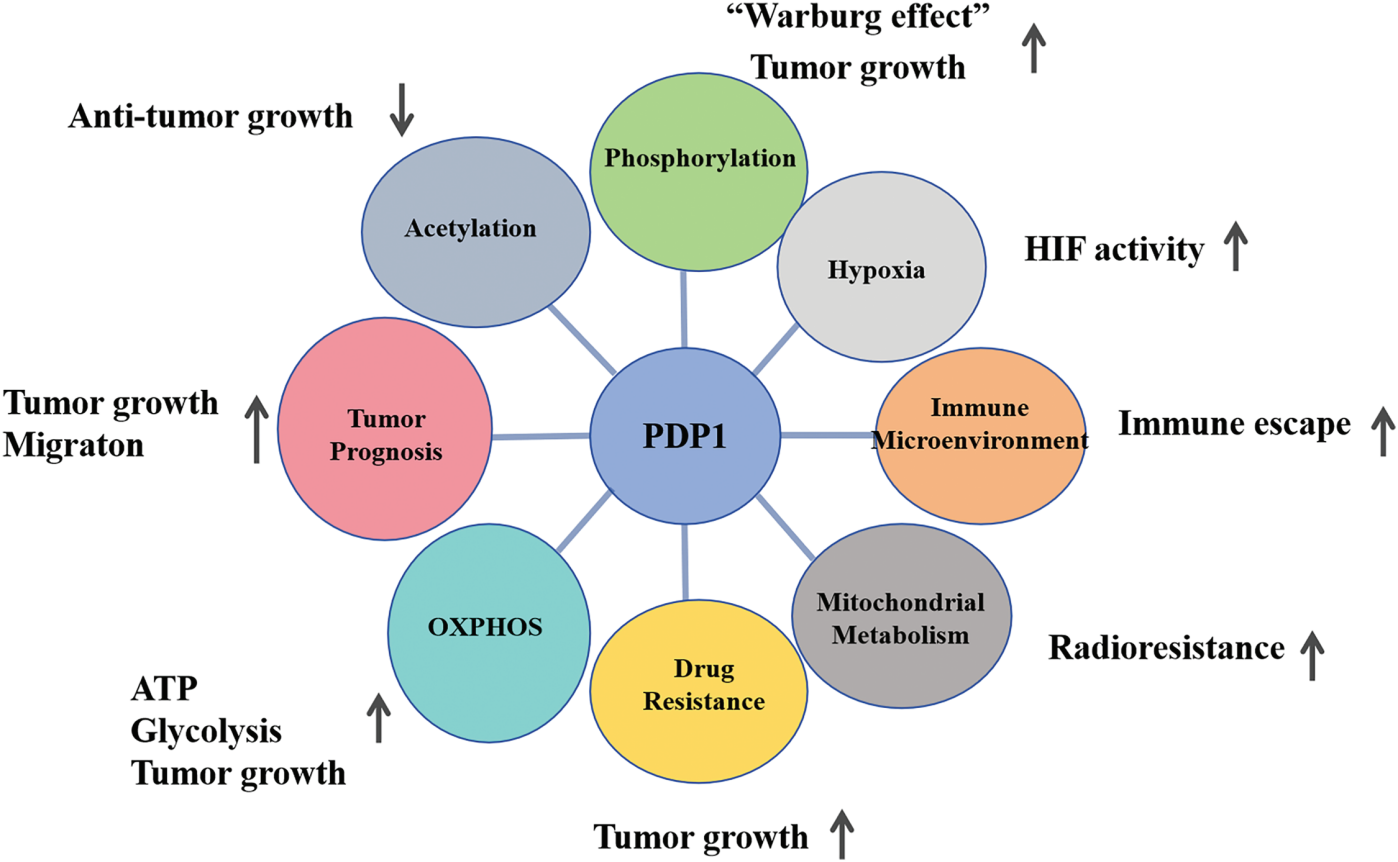
The diagram of metabolic pathways is associated with PDP1 and their role in tumors. PDP1 significantly influences tumor behavior by promoting proliferation and migration, enhancing resistance to radiotherapy, boosting metabolism, and facilitating immune evasion (Conducted by Figdraw).

The catalytic subunit of pyruvate dehydrogenase phosphatase is known as PDP1. The human PDP1 gene encodes this protein, which belongs to the protein phosphatase 2C (PP2C) superfamily. PDP1 is a non-monomeric phosphatase located in the mitochondrial matrix [83]. In physiological terms, PDP1 serves as a key regulator of PDC, positively influencing its catalytic activity through the removal of phosphate groups from serine residues [84]. Once PDP1 activates PDC, the enzyme irreversibly catalyzes pyruvate into acetyl-CoA, which is the primary substrate for energy production in cellular metabolism, for instance, provides fuels for the tricarboxylic acid (TCA) cycle, through the TCA cycle supplies energy for OXPHOS and furnishes raw materials for protein, lipid, and nucleotide synthesis [6]. (Figs. 3 and 4)

#### PDP1 and acetylation

Acetylation, catalyzed by acetyltransferase or occuring through non-enzymatic, was transferred an acetyl group to either the lysine residue or the N-terminus of a protein. In lung cancer cells (H1299), increased lysine acetylation of PDP1 results in decreased phosphatase activity, which suggests that this acetylation inhibits PDP1’s function. Mass spectrometry experiments identified the K202 site on PDP1 as the lysine acetylation site, and changes in PDP1 enzyme activity were linked to variations in lysine acetylation levels. The elimination of lysine acetylation on PDP1 induces metabolic alterations that heighten reliance on OXPHOS, which negatively affects cell proliferation in hypoxic conditions. In H1299 cells, consistent downregulation of acetyl-CoA acetyltransferase 1 (ACAT1) leads to increased PDP1 activity and reduced acetylation level of PDP1 at K202. In H1299 cells, consistent downregulation of ACAT1 leads to increased PDP1 activity and reduced acetylation levels at PDP1 K202. The study demonstrated that ACAT1 primarily signals through the PDP1/PDHA1 axis to promote glycolysis. While sirtuin 3 (SIRT3) directly deacetylates (pyruvate dehydrogenase E1 subunit alpha 1) PDHA1 and PDP1, working in concert with ACAT1 to regulate their deacetylation and acetylation, indicating that the tyrosine kinase signaling pathway regulates mitochondrial ACAT1 and SIRT3 accessibility to PDC by modulating PDP1 [85].

#### PDP1 and phosphorylation

Phosphorylation is the addition of a phosphate group to either metabolic intermediates or proteins. The phosphorylation of various tyrosine residues inhibits PDP1 through independent mechanisms, which promotes the “Warburg effect”. *In vitro* studies using fibroblast growth factor receptor 1 (FGFR1) demonstrated the phosphorylation of PDP1 at the Y79, Y94, and Y381 sites. Mutation analysis produced various PDP1 mutants, by replacing the identified phosphorylated at specific tyrosine residues with phenylalanine (Y/F). Researchers discovered that phosphorylation at the Y381 site of PDP1 regulates lysine acetylation by changing the expression of acetyl-CoA acetyltransferase1 (ACAT1) and PDP1 in PDC and contributing to tumor cell metabolism and proliferation [86]. Research on tumor cells demonstrated that the phosphorylation-deficient PDP1 Y94F mutant increased OXPHOS in mice, reduced cell proliferation under hypoxic conditions, and consequently slowed tumor growth. This study found that phosphorylation at different tyrosine residues inhibits PDP1 through independent mechanisms, coordinating the regulation of PDC activity to promote the “Warburg effect”. PDP1 phosphatase activity depends on its binding to the L2 domain of dihydrolipoyl acetyltransferase in PDC, a process that is mediated by the covalent attachment of lipoic acid to this domain, which typically requires calcium. In the absence of calcium, PDP1 cannot form a stable complex with dihydrolipoyl acetyltransferase, significantly reducing its catalytic activity [34]. Phosphorylation of tyrosine(Tyr)-94 may affect PDP1’s activity by diminishing its ability to bind to lipoic acid and dihydrolipoyl acetyltransferase [86].

#### PDP1 and hypoxia

Hypoxia is a condition where tissues undergo abnormal changes in metabolism, function, and structure due to insufficient oxygen supply or impaired oxygen utilization. Silencing PDP1 reduces hypoxia-inducible factor (HIF) activity and the expression of HIF-dependent genes. However, overexpressing this protein increases HIF activity in low-oxygen conditions. The levels or activity of PDP1 do not alter HIF-1α protein levels, its nuclear accumulation, or its interactions with aryl hydrocarbon receptor nuclear translocator (ARNT) and nucleophosmin 1 (NPM1). The downregulation of PDP1 did not alter the mRNA levels of HIF-1α and HIF-2α under both normoxic and hypoxic conditions. This finding suggests that PDP1 does not influence HIF mRNA levels, consistent with previous results. The research revealed that PDP1 activates HIF-1 by strengthening its binding to target gene promoters [84]. This study indicates that PDP1 may be involved in a regulatory feedback mechanism that enhances HIF transcription during hypoxia. It achieves this by maintaining pyruvate dehydrogenase (PDH) activity, acetyl-CoA levels, and histone acetylation at the promoters of hypoxia-target genes. Therefore, under hypoxic conditions, the depletion of PDP1 negatively impacts the binding of HIF-1 to known promoters and acetylation. This reduction in acetylation may be due to low levels of acetyl-CoA, but it could also be a result of weaker HIF-1 binding, as the acetylation inhibition may also affect HIF-1 itself or its transcriptional coactivators. On the other hand, when PDP1 and PDH are overactivated under hypoxic conditions, the stimulation of HIF-1 activity and the induction of PDK1 can reduce the conversion of pyruvate to acetyl-CoA, thereby maintaining glycolysis and lactate production [87].

#### PDP1 and immune microenvironment

The tumor immune microenvironment is critically important, as it directly influences tumor development and determines clinical outcomes [88–90]. The immune characteristics of the tumor microenvironment are currently recognized as one of the ten hallmarks of cancer, and they could significantly influence the effectiveness of radiotherapy and chemotherapy [91–93]. Analyzing the types and distribution of immune cells in the tumor microenvironment, along with establishing an effective immune evaluation system, carries significant clinical implications. In the tumor microenvironment, the dynamic network is primarily composed of stromal and immune cells found in tumor tissues [94–96]. By analyzing the correlation between different types of immune cell infiltration and overall survival in breast cancer patients using bioinformatics methods, it was found that M1 macrophages and CD8+ T cells positively correlated with the 5-year survival rate of breast cancer susceptibility gene (BRCA) mutation patients, while M2 macrophages negatively correlated with this metric. B cells, T cells, memory CD4+ T cells, and follicular helper T cells were not correlated with the 5-year survival rate of breast cancer patients. *In vitro* studies revealed that increased levels of PDP1 expression reduced the invasion of CD8+ T cells in breast cancer [97]. Macrophage activation is a key event in the pathogenesis of various inflammatory diseases. The Notch signaling pathway is crucial for activating pro-inflammatory macrophages. Salidroside, a key bioactive component of *Rhodiola rosea*, has been identified for its potential role in modulating macrophage activity. *In vitro* studies found that the expression of Notch1 and PDP1 significantly increased in THP-1 macrophages after simultaneous stimulation with alcohol and lipopolysaccharide. The addition of salidroside then reduced the Notch-dependent induction of PDP1. This indicates that Notch1 enhances PDP1 expression, which enhances glycolysis and glucose flow into the tricarboxylic acid cycle via PDP1-mediated PDH activation [98].

#### PDP1 and mitochondrial metabolism

Mitochondrial dysfunction may activate mitochondrial retrograde signaling (mito-RTG), which could influence regulating tumor metabolic reprogramming and cell sensitivity to radiation. Research indicates that phosphorylated PDH and PDK1, which play a role in aerobic glycolysis, are positively linked to radioresistance, however, their initiation and function within the mitochondrial retrograde signaling pathway remain unclear. Further genomic analysis showed that complex I components were widely downregulated in mitochondrial dysfunction models. High levels of phosphorylated PDH were found in cells deficient in complex I, which induced radioresistance. Mechanistically, defects in complex I result in a loss of membrane potential in the respiratory chain, which decreases mitochondrial Ca^2+^ levels and leads to the inactivation of PDP1. Complex I defects lead to decreased levels of PDH in both the cytoplasm and nucleus via the Ca^2+^-PDP1-PDH axis, while lower PDH levels in the nucleus promote DNA damage repair responses through reduced histone acetylation [99,100]. At the same time, overexpression of NADH: ubiquinone oxidoreductase core subunit S1 (NDUFS1) can enhance the activity of complex I, reverse glycolysis and render tumor cells re-sensitive to radiation, A common metabolic feature in glioblastoma cell lines is reduced mitochondrial reserve capacity. This phenotype is associated with RAS-mediated signaling pathways that inhibit PDH activity by reducing PDP expression [101]. The Cancer Genome Atlas (TCGA) database indicates that PDP1 levels are significantly suppressed in glioblastoma. Research using a mouse model of glioblastoma indicated that restoring PDP1 expression reduces tumorigenicity. Moreover, the RAS signaling pathway regulates PDH activity through PDP1, without affecting PDK1 [102].

#### PDP1 and drug resistance

The high metabolic sensitivity in acute myeloid leukemia is a fundamental determinant of both treatment persistence and the emergence of resistance. Approximately 20% to 30% of patients with acute myeloid leukemia carry activating mutations in fms related receptor tyrosine kinase 3 (FLT3), particularly the FLT3-ITD mutation, which is a critical target for therapy. In addition to activating mutations, the molecular factors that affect FLT3 inhibitor response and resistance are not well understood. Like most tumor cells, acute myeloid leukemia cells consume a large amount of glucose to obtain energy for cell proliferation [103–105]. However, enhanced lactate glycolysis does not necessarily come at the expense of diminished mitochondrial pyruvate metabolism [106–108], which is considered an important metabolic feature of tumors, especially acute myeloid leukemia [14,109–111]. Mitochondrial OXPHOS has recently attracted attention [112,113]. Studies on FLT3-ITD metabolism in acute myeloid leukemia found that PDP1 gene knockdown reduced cellular respiration, thereby weakening the proliferation of FLT3-ITD-positive cells, even under hypoxic conditions, FLT3-ITD-positive cells continued to rely on PDP1 and exhibited rapid, PDP1-dependent recovery of respiratory capacity during reoxygenation. This finding indicates that FLT3-ITD regulates the expression of PDP1, and PDP1 may be a key metabolic regulator for enhancing oxidative glycolysis and resistance [114].

#### PDP1 and oxidative phosphorylation

Oxidative phosphorylation is a biochemical process that occurs in the inner membrane of mitochondria in eukaryotic cells and in the cytoplasm of prokaryotic cells. This coupled reaction harnesses energy released from nutrient oxidation to allow the respiratory chain to produce ATP from ADP and inorganic phosphate. In studies of acute myeloid leukemia, PDP1 is essential for FLT3-ITD cells under hypoxia, as it allows for a rapid transition to OXPHOS when oxygen becomes available; this transition is linked to significant upregulation of PDP1 upon inhibition of the FLT3-ITD signaling pathway, which results in a distinct OXPHOS metabolic profile with provides cytotoxic protection. Regardless of PDP1’s function, its role in maintaining acute myeloid leukemia cell survival depends on its ability to enhance the OXPHOS activity [114]. Through studies on diffuse large B-cell lymphoma, it was found that in ibrutinib-resistant cells, early growth response factor 1 (EGR1) increased its binding to the PDP1 promoter and transcription start site region while downregulation of EGR1 led to a reduction in PDP1 mRNA and protein expression levels, which in turn increased PDH phosphorylation levels. High expression of PDP1 was noted in ibrutinib-resistant mantle cell lymphoma cell lines. Knocking out EGR1 significantly reduced PDP1 expression, subsequently leading to a significant inhibition of PDH activity. Mechanistically, the self-regulation of transcription factor 4 (TCF4) leads to the overexpression of EGR1, which mediates metabolic reprogramming to OXPHOS through the PDP1 activation. PDP1 is a phosphatase that can dephosphorylate and activate the E1 component of the large PDC. Therefore, EGR1-mediated PDP1 activation increases intracellular ATP production, providing sufficient energy to enhance the proliferation and survival of ibrutinib-resistant lymphoma cells [115].

#### PDP1 and tumor prognosis

Bioinformatics analysis of ovarian cancer data indicates a high expression of PDP1 in ovarian cancer tissues. This expression is linked to poor prognosis and unfavorable clinical characteristics. PDP1 is associated with immune cells, and its expression is negatively correlated with the half-inhibitory concentrations of bleomycin and gemcitabine, but positively correlated with cisplatin [116]. PDP1 enhances the proliferation, invasion, and migration of ovarian cancer cells. Pathways associated with PDP1 are mainly enriched in the interleukin-6 (IL-6)/Janus kinase (JAK)/signal transducer and activator of transcription 3 (STAT3) signaling pathway, interferon-response, apoptosis, adipogenesis, Kirsten rat sarcoma viral oncogene homolog (KRAS) signaling pathway, and interleukin-2 (IL-2)/signal transducer and activator of transcription 5 (STAT5) signaling pathway. Preliminary research shows that the PDP1 gene is overexpressed in breast cancer tissues and correlates with poor prognosis, Validation through *in vivo* and *in vitro* experiments further confirmed that PDP1 contributes to cancer progression by regulating STAT3 phosphorylation levels [117]. Further research on brain metastasis in breast cancer has confirmed that overexpressing STAT3 significantly restores the proliferative ability of triple-negative breast cancer (TNBC), STAT3 is closely related to the autophagy process and participates in the occurrence of brain metastasis in TNBC by regulating the expression of autophagy-related genes [118]. In pancreatic cancer tissue studies, it was found that PDP1 is highly expressed and is associated with poor patient prognosis [5]. PDP1 promotes the proliferation, invasion, and migration of pancreatic cancer cells. Additionally, it accelerates the production of ATP within these cells, which provides sufficient energy for tumor cells and supports rapid tumor progression. PDP1 overexpression downregulates adenosine monophosphate (AMP)-dependent protein kinase, resulting in the mechanistic target of rapamycin kinase (mTOR) activation and promoting tumor progression [119]. Bioinformatics data analysis found that PDP1 is highly expressed in breast cancer tissues, and PDP1 expression is positively correlated with N-stage and negatively correlated with CD8+ T cell invasion [96]. In prostate cancer tissues, the PDP1 protein is highly expressed, and elevated PDP1 levels are associated with enhanced mitochondrial oxygen consumption from glucose and glutamine [6]. Increased levels of PDP1 in KRAS mutant colorectal cancer (CRC) cells and tissues are linked to a worse prognosis. Both *in vivo* and *in vitro* studies show that PDP1 enhances the malignancy of KRAS mutant colorectal cancer cells. Subsequent experiments reveal that PDP1 serves as a scaffold. It promotes colorectal cancer progression by enhancing the interaction between BRAF and Mitogen-activated protein kinase kinase 1 (MEK1), leading to the activation of the mitogen-activated protein kinase (MAPK) signaling pathway [120]. Analysis of PDP1 expression in pan-cancer found that PDP1 is widely expressed in epidermal growth factor-activated cells and various human malignant tumor cells. PDP1 expression correlates with survival outcomes across various cancers, and tumors with low PDP1 levels tend to have relatively better survival outcomes. Tumors with low PDP1 expression show increased immune activity, characterized by CD8+ T cell infiltration. Single-cell transcriptomic analysis indicates that hsa-miRNA-655-3p positively regulates PDP1 specifically in low-grade gliomas. hsa-miRNA-135b-5p positively regulates PDP1 in low-grade gliomas, while negatively regulating PDP1 in head and neck squamous cell carcinoma and pancreatic cancer [121].

### Pyruvate dehydrogenase phosphatase regulatory subunit 2 in malignant tumors

The pyruvate dehydrogenase phosphatase regulatory subunit, known as PDP2, is encoded by the human PDP2 gene, which belongs to the protein phosphatase 2C (PP2C) superfamily and is located in the mitochondrial matrix. Studies have shown that PDP2 can inhibit hepatitis B virus (HBV) transmission by removing phosphorylation groups from two phosphorylation sites, Ser162 and Thr160, on the core protein [122]. In addition, PDP2 has been found to regulate lipid metabolism, cellular senescence, and the differentiation and functional activity of Th17 cells [6,123,124]. These findings indicate that PDP2 has a series of roles beyond energy metabolism. Therefore, further research is essential to clarify the role of PDP2 in the progression of breast cancer. PDP2 is highly expressed in breast cancer tissues and is significantly correlated with patient prognosis. PDP2 induces ferroptosis in tumor cells by dephosphorylating and inhibiting the Acyl-CoA Synthetase Long-Chain Family Member 4 (ACSL4) activity [125]. Under normoxic and CoCl_2_-induced hypoxic conditions, silencing the gastrin-releasing peptide receptor (GRP-R) caused a decrease in HIF-1α expression, which blocked vascular endothelial growth factor (VEGF) expression and secretion and led to lower PDK4 levels and higher PDP2 mRNA levels, indicating that GRP-R regulates glucose metabolism in neuroblastoma by modulating HIF-1α, PDK4, and PDP2 [126]. In prostate cancer patients, the metabolic gene PDP2 associated with metastasis was identified through the analysis of circulating tumor cells (CTC) biofunctional [127]. In A549 non-small cell lung cancer (NSCLC) cell line, muscle larva excretory/secretory products (ML-ESPs) induced the transcription of glucose metabolism reprogramming-related genes, and PDP2 was significantly upregulated [128]. Bioinformatics analysis demonstrated that lung cancer cells exposed to nitric oxide (NO) experienced changes in transcription factors (TFs) and epigenetic modifications (histone modification and miRNA), which significantly reduced PDP2 levels [129]. PDP2 plays an important role in tumor metabolic reprogramming, regulating the growth and survival of cancer cells.

## Conclusion

Mitochondrial pyruvate dehydrogenase phosphatase metabolic abnormalities have been widely recognized as an important marker of the occurrence and development of malignant tumors. Through in-depth research on this metabolic pathway, the characteristics of cancer cell metabolic reprogramming have been revealed, offering new insights into the biological basis of malignant tumors. Although specific mechanisms and roles may vary across studies, all research underscores the central role of this enzyme in tumor metabolism. In the current research, although existing results provide us with many important clues, there are still many unknown mechanisms to explore. Future research should focus on refining molecular mechanism analysis and exploring its clinical applications. Therefore, identifying new small molecule inhibitors and biologics, along with their clinical applications, is an important research direction. A phase II trial demonstrated that dichloroacetate (DCA) significantly reduces pyruvate levels in patients with head and neck carcinoma, but its safety profile is still unclear, with reports of neurotoxicity and potential liver damage limiting its clinical use [130]. The PDH inhibitor CPI-613 has advanced to Phase III clinical trials for pancreatic cancer treatment, showing encouraging results in patient responses [131]. Although there are no current reports about drug design and clinical research related to PDP1. However, this research provides critical insights that facilitate the development of drugs targeting PDP1. Additionally, strengthening the connection between basic research and clinical applications, and promoting translational medicine in developing new therapies, will lead to more effective personalized treatment plans.

In summary, the role of mitochondrial PDP in malignant tumors is multifaceted. Its potential mechanisms within acetylation, phosphorylation, hypoxia, immune infiltration, mitochondrial metabolism, drug resistance, oxidative phosphorylation, and tumor prognosis. This article reviews the effective integration of tumor metabolic reprogramming with clinical issues by PDP1, highlighting its potential implications for future cancer treatments. Future research should connect basic science with clinical applications to advance new treatment strategies. A thorough understanding of the mechanisms behind this metabolic abnormality is expected to lead to significant advancements in cancer prevention, early diagnosis, and personalized treatment. Therefore, continuing to explore this field not only has important scientific value but also provides practical help for improving patient survival outcomes.

## Data Availability

Data will be made available on request.
